# Efficacy and Safety of Capecitabine for Triple-Negative Breast Cancer: A Meta-Analysis

**DOI:** 10.3389/fonc.2022.899423

**Published:** 2022-07-07

**Authors:** Xueqiong Xun, Qinguang Cao, Pan Hong, Saroj Rai, Yeming Zhou, Ruikang Liu, Huiyong Hu

**Affiliations:** ^1^ Department of Thyroid and Breast Surgery, First People’s Hospital of Qujing, Qujing, China; ^2^ Department of Orthopaedic Surgery, Union Hospital, Tongji Medical College, Huazhong University of Science and Technology, Wuhan, China; ^3^ Department of Orthopaedics and Trauma Surgery, Blue Cross Hospital, Kathmandu, Nepal; ^4^ Department of Orthopaedics and Trauma Surgery, Karama Medical Center, Dubai, United Arab Emirates; ^5^ Basic Medical School, Tongji Medical College, Huazhong University of Science and Technology, Wuhan, China; ^6^ Department of Endocrinology, Union Hospital, Tongji Medical College, Huazhong University of Science and Technology, Wuhan, China

**Keywords:** triple-negative breast cancer (TNBC), capecitabine, xeloda, chemotherapy, meta-analysis

## Abstract

**Background:**

Triple-negative breast cancer (TNBC) is the most aggressive subtype of breast cancer with limited treatment options and poor prognosis. Capecitabine, as a novel adjuvant chemotherapy for TNBCs, remains controversial. Therefore, we conducted this meta-analysis to assess the efficacy and safety of capecitabine for early-stage TNBCs combined with neo-/adjuvant chemotherapy.

**Methods:**

We searched Medline, Embase, Web of Science, and Cochrane databases updated on Mar 18, 2022 for relevant RCTs. In all, 11 RCTs with 5,175 patients were included. We used hazard ratios (HRs) and odds ratios (ORs) to assess the differences between disease-free survival (DFS), overall survival (OS), and adverse events.

**Results:**

Our study demonstrated significance differences in both DFS and OS (DFS: HR=0.77; 95% CI 0.68–0.86; OS: HR=0.73, 95% CI 0.63–0.85). In subgroup analysis, the lower dosage group showed higher DFS (HR=0.79, 95% CI 0.69–0.91), higher frequency (HR=0.72, 95%CI 0.62–0.83), and adjuvant chemotherapy (HR=0.74, 95% CI 0.65–0.84). However, capecitabine was also associated with a higher risk of diarrhea (OR=3.10, 95% CI 2.32–4.15), hand–foot syndrome (OR=25.79, 95% CI 15.32–43.42), and leukopenia (OR=2.08, 95% CI 1.13–3.84).

**Conclusion:**

The addition of capecitabine to early-stage TNBC patients receiving standard adjuvant chemotherapy showed significant DFS and OS improvement with tolerable adverse events. The lower dosage and higher frequency of capecitabine combined with adjuvant chemotherapy demonstrated a better survival outcome.

## Introduction

Breast cancer is the second most common cancer in women (1). Waks et al. reported in 2017 that nearly 12% of women would be diagnosed with this fatal disease in the US during their lifetime (1). Triple-negative breast cancer (TNBC), devoid of estrogen receptors (Ers), progesterone receptors (PRs), and human epidermal growth factor receptor 2 (HER-2), is the most aggressive subtype with limited treatment options and poor prognosis (2). The major components of TNBC in molecular assays are normal breast-like tumors, basal-like tumors, and newly found claudin-low molecular subtypes. BRCA1-deficient breast tumor is also included in TNBC (3). During the first 3 years after the diagnosis of TNBCs, there existed a high recurrence rate with site-specific distribution (4, 5).

Patients with TNBCs could hardly be treated with endocrine therapy or any other treatment targeting those three receptors (6). The standard chemotherapy for early-stage TNBC consists of different combinations of anthracycline, taxane, cyclophosphamide, and fluorouracil. Besides standard chemotherapy, the addition of any other drugs was regarded as a new regimen (7). However, most evidence supporting chemotherapy is derived from retrospective analyses of clinical trials before 2010 (8). Besides, the 10-year risk of relapse of early TNBC is still up to 20%–40% (9), and women with TNBCs are diagnosed at a younger age (4). Therefore, updating neo-/adjuvant chemotherapy drugs or regimens is crucial to improve the therapeutic effect.

Capecitabine is an oral prodrug of fluorouracil that has been proven to be effective in treating gastric cancer and advanced breast cancer (10, 11). Nowadays, several randomized clinical trials (RCTs) have evaluated the efficacy and safety of capecitabine-based neoadjuvant and adjuvant chemotherapy in early-stage triple-negative breast cancer. Still, the result remained heterogeneous (10, 12, 13). Some meta-analyses had analyzed several RCTs associated with capecitabine, but TNBCs were treated as a subgroup in these studies (9). Therefore, we conducted this meta-analysis to evaluate the efficacy and safety of capecitabine for TNBC treatment.

## Method

### Search Strategy

This review followed the guidelines of Preferred Reporting Items for Systematic Reviews and Meta-Analyses (PRISMA) (14), and the protocol was registered in PROSPERO before the literature search. Two independent reviewers (QC and PH) searched Medline, Embase, Web of Science, and Cochrane databases updated on March 18, 2022 for RCTs. To expand the search range, we used the keywords “breast cancer,” “breast neoplasms,” “Capecitabine,” “Xeloda,” and “adjuvant chemotherapy.” The detailed search strategy used for the Medline database is available in the supplementary material (see **Supplementary Table S2**). Clinicaltrials.gov was also searched for completed but unpublished RCTs with published results. We used truncated terms for all fields and categorized study types as clinical trials or randomized controlled trials. Two researchers (SR and YZ) independently screened the titles and abstracts, and articles meeting inclusion criteria were accessed for full-text review. Reference lists of eligible reviews and trials were searched for additional citations.

### Inclusion Criteria

Inclusion criteria were as follows: (1) phase III RCTs; (2) experiment group received neo-/adjuvant chemotherapy with capecitabine, while the control group received chemotherapy without capecitabine; and (3) RCTs with available data of hazard ratios (HRs) of 95% confidence intervals (CI) for DFS and OS. In addition, only RCTs published in English were included, and there was no restriction on age, sex, nationality, and race.

### Data Extraction

Two researchers (RL and YZ) independently extracted data from eligible articles and aggregated the results. The divergences were settled to consensus by consulting a third reviewer, HH. The information we extracted included characteristic of the study (author, year of publication, journal, publication type, objective, type of disease, inclusion criteria, exclusion criteria, administration method, exposure, follow-up, and funding source), characteristic of the patient (number of participates and age), and the outcomes. Outcomes were classified as primary outcomes and secondary outcomes. Primary outcomes included DFS and OS change from baseline. Recurrence-free survival (RFS), defined as the survival time between the dates of randomization and detection of invasive breast cancer recurrence and metastasis or death if the patient died prior to recurrence or metastasis, was similar to the DFS in other studies (10). Therefore, we aggregated RFS and DFS together for later pooled trials. Secondary outcomes included the grade 3–5 drug-related adverse events, including neutropenia, diarrhea, fatigue, and hand–foot syndrome. For studies that reported many outcomes, we recruited the latest one. If the data were incomplete, the corresponding author would contact the author by email and invite them to send additional information for further research.

### Quality Assessment

Cochrane Risk of Bias Assessment Tool (CROBAT) was used by two researchers (XX and QC) to assess the quality of included studies independently. CROBAT included “random sequence generation,” “allocation concealment,” “blinding of participants and personnel,” “blinding of outcome assessment,” “incomplete outcome data,” “selective reporting,” and “other bias” (see **Supplementary Table S3**). Each question had three answers: “low risk,” “moderate risk,” and “high risk.” Researchers would assess the risk level of RCTs according to the published information. The decision was reached by consulting a third reviewer, PH, in the case of disagreements or failed consensus. Publication bias was evaluated by funnel plots, and p ≤ 0.05 was considered a statistically significant risk of bias. Small-study effects that led to potential reporting or publication bias could be calculated by Egger’s test. We used the Grading of Recommendations, Assessment, Development, and Evaluation (GRADE) tool to evaluate the quality of evidence for each outcome. The GRADE tool classified evidence of outcomes into “High,” “Moderate,” “Low,” and “Very low.” Each assessment could reduce or promote the level of quality. Specific rules are explained in **Supplementary Table S3**.

### Statistical Analysis

The HRs and 95% CIs for DFS and OS were collected, and they were weighted and combined by the generic inverse variance method (15). Heterogeneity in the result of meta-analysis was assessed using Cochrane Q and I^2^ statistics with appropriate analysis models. When p ≤ 0.05 or I^2^ > 50%, the random effects model was used, and when p > 0.05 or I^2^ < 50%, the fixed effects model was used (16), and dichotomous data was calculated by odds ratio (OR) with 95% CIs.

Subgroup analyses were carried out according to the dosage of capecitabine, the number of cycles using capecitabine, neoadjuvant or adjuvant chemotherapy, lymph node positivity or negativity, and menopausal status. Sensitivity analysis was performed in the meta-analysis by excluding each study once at a time to check whether the effectiveness of the outcome was determined by individual studies. All statistical analyses were performed using Review Manager 5.3 and STATA 16.0.

## Result

### Characteristics of the Studies

**Figure 1:**
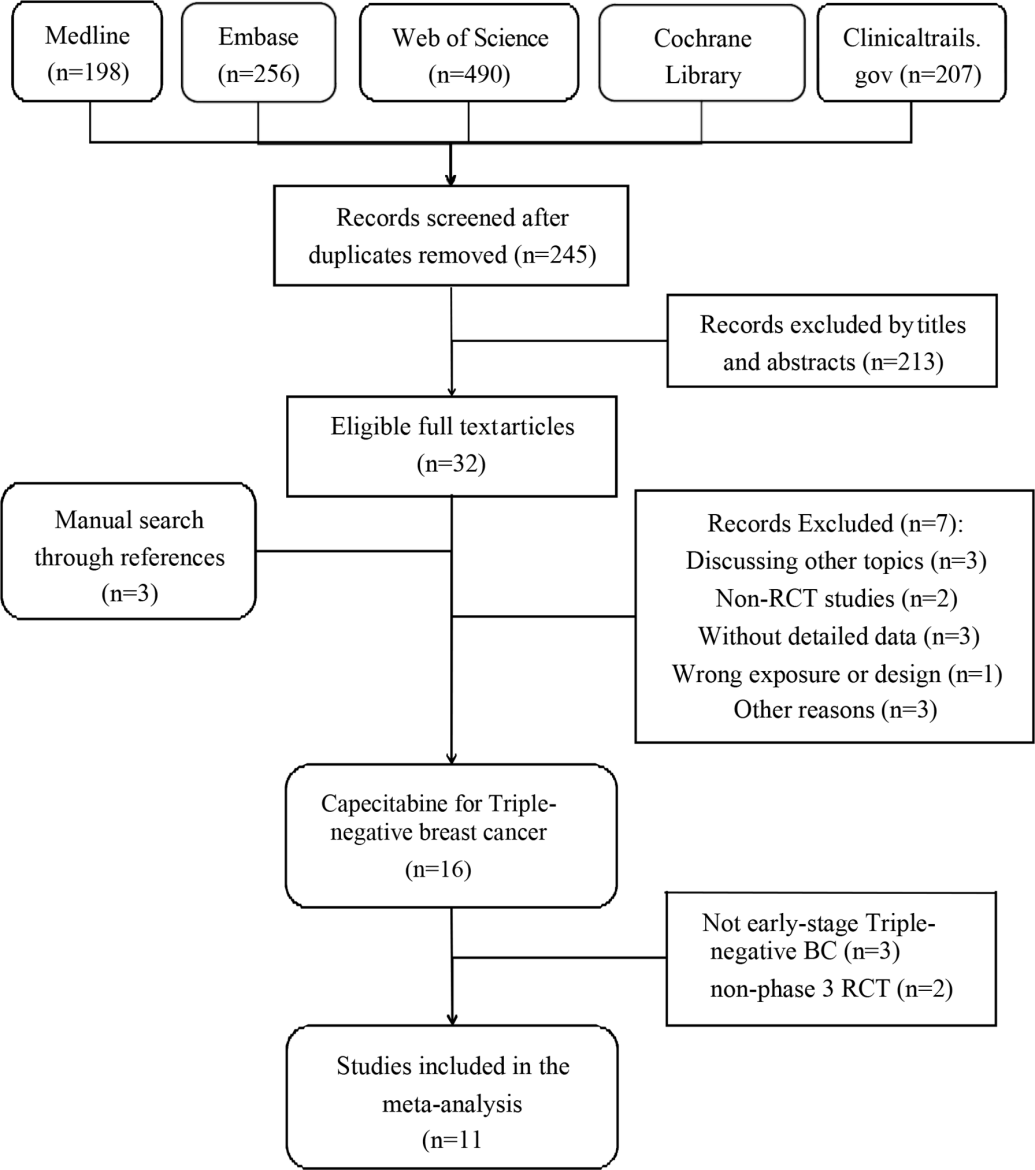
Flowchart of selection of included studies.

We included 11 RCTs with 5,175 female patients in our meta-analysis (**Supplementary Table S1**). **Figure 1** demonstrates the flowchart of the search process of our study. Among these RCTs, four RCTs only discussed TNBC (12, 17–19), while TNBC patients in other seven RCTs were regarded as a subgroup (10, 13, 18, 20–23). We used CROBAT to assess the quality of included studies. **Supplementary Table S4** demonstrates the risks of bias in our study that all RCTs are double-blinded and randomized.

**Figure 2:**
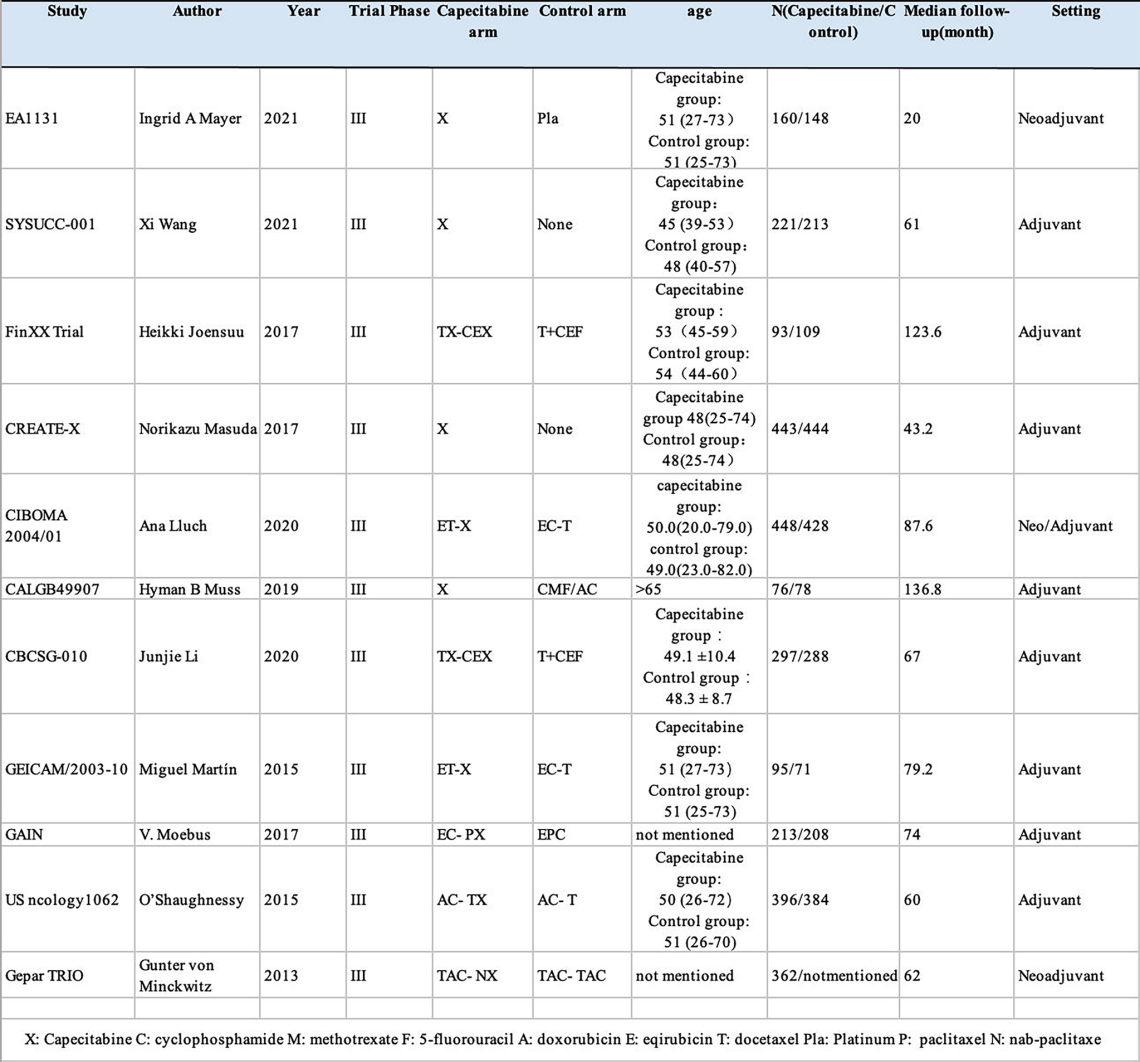
The baseline characteristics.

Eight RCTs reported the HRs and 95% CIs of DFS, whereas in the Fin XX trial, the CALGB49907 trial, and the EA1131 trial, they presented survival data of RFS instead of DFS. Moreover, nine RCTs reported the OS HRs and 95% CIs for OS except for the Gepar TRIO trial and GEICAM/2003-10 trial. The research features are shown in **Figure 2**.

### DFS and OS

**Figure 3:**
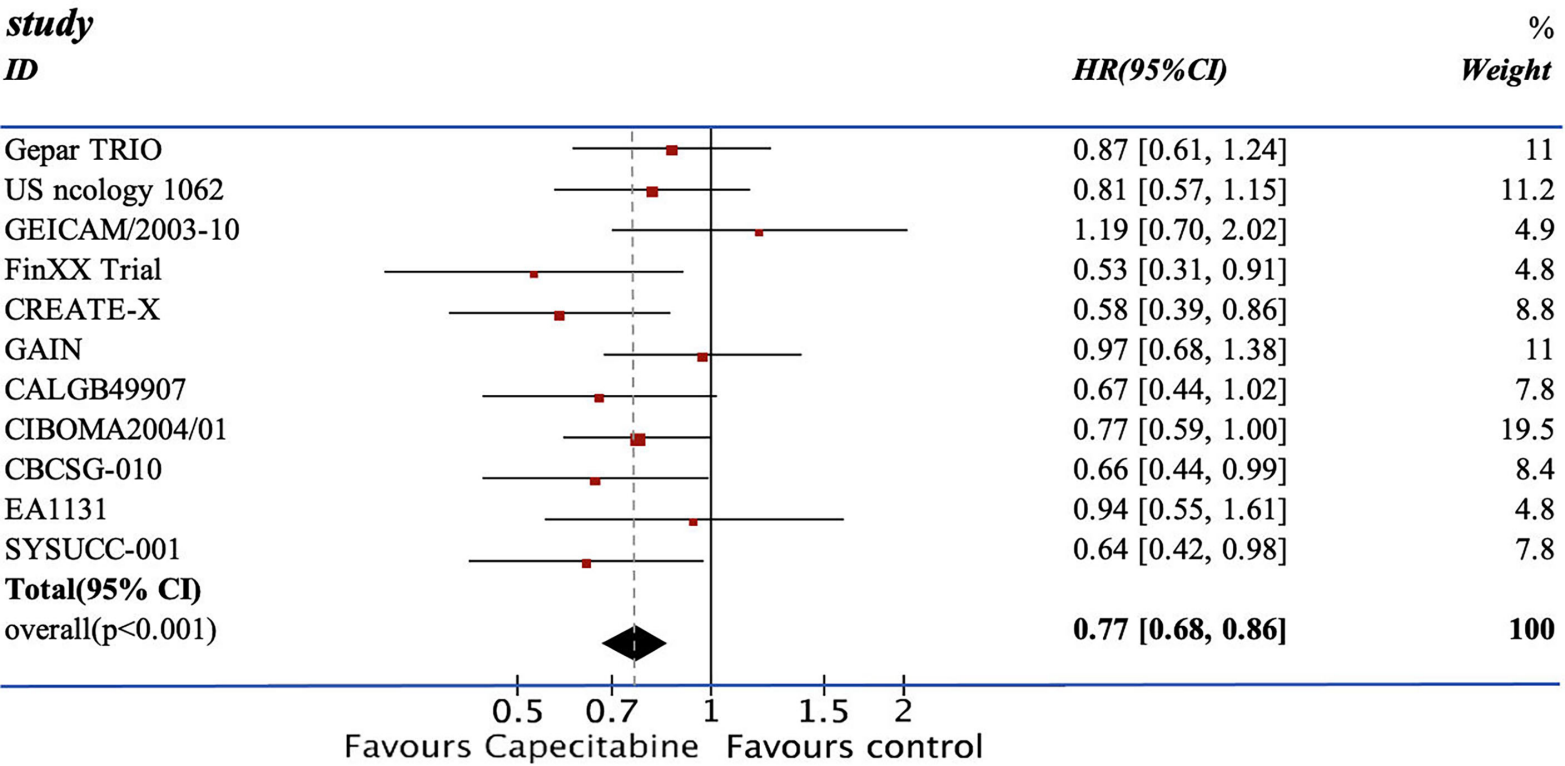
The result of the DFS.

**Figure 4:**
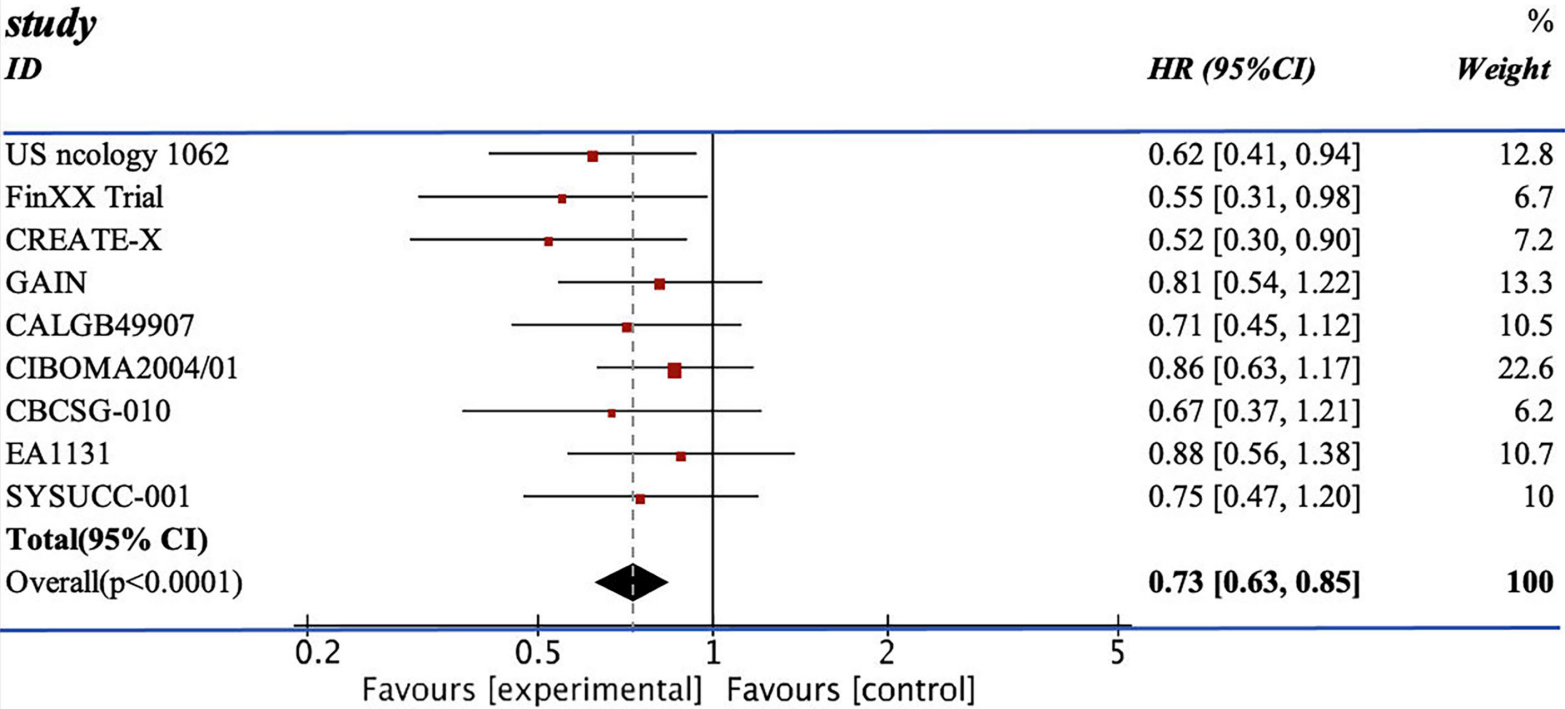
The result of the OS.

We pooled the HRs of 11 RCTs for DFS and OS. From the data available, 2,804 patients from the experimental group were treated with chemotherapy containing capecitabine, and the patients in the control group were assigned to the therapeutic method without capecitabine. The addition of capecitabine was significantly associated with the improved DFS (HR=0.77; 95% CI, 0.68–0.86) with low heterogeneity (I^2 =^ 7%). They also showed apparent increase in OS (HR=0.73, 95% CI 0.63–0.85) with low heterogeneity (I^2 =^ 0%) (see **Figures 3**, **4**).

### DFS Subgroup Analysis

#### Dosage

We took 1,000 mg as a dividing line. Eight studies applied ≥1000 mg capecitabine as treatment (12, 18–24), and the other three RCTs used <1,000 mg capecitabine (10, 13, 17). DFS was significantly improved in the group with ≥1,000 mg capecitabine (HR=0.79, 95% CI 0.69–0.91) with low heterogeneity, and in the <1,000 mg capecitabine group (HR=0.69; 95% CI, 0.54–0.88) with insignificant subgroup difference (p=0.32) (see **Supplementary Figure S1**).

#### Neo-/Adjuvant Chemotherapy

Capecitabine was regarded as adjuvant chemotherapy in eight RCTs (10, 13, 17–22) and neoadjuvant chemotherapy in two RCTs (12, 23). The CIBOMA 2004/01 trial used capecitabine both as adjuvant and neoadjuvant chemotherapy. Based on the data assembled, the capecitabine in the neoadjuvant group (HR=0.92, 95% CI 0.71–1.19) did not show a significant difference in DFS. In contrast, patients receiving capecitabine as adjuvant chemotherapy obtained a higher DFS (HR=0.74, 95% CI 0.65–0.84) without significant subgroup difference (p=0.15) (see **Supplementary Figure S2**).

#### Cycles

Moreover, there were seven RCTs (12, 13, 17–20, 24) that adopted ≥6 cycles and four (10, 21–23) RCTs that adopted <6 cycles of capecitabine for treatment. From the available information about TNBC, there were 1,151 cases treated with <6 cycles of capecitabine, and 4,024 cases underwent ≥6 cycles of capecitabine. A significant improvement of DFS was found in the ≥6 cycles group (HR=0.72, 95% CI 0.62–0.83) but not in the <6 cycles group (HR=0.88, 95% CI 0.71–1.08) with unapparent subgroup difference (p=0.23) (see **Supplementary Figure S3**).

#### Other Subgroups

We also conducted analysis on the other three subgroups, which resulted in a significant subgroup difference: (1) menopausal status, premenopausal women had a higher DFS (HR=0.72, 95%CI 0.50–1.05) (see **Supplementary Figure S4**); (2) Ki-67, the patients with Ki-67<30% showed a better survival result (HR=0.53, 95%CI 0.29–0.98) (see **Supplementary Figure S5**); (3) nodal status, there was not any apparent difference between positive lymph node (HR=0.68, 95%CI 0.52–0.89) and negative lymph node (HR=0.68, 95%CI 0.50–0.92) (see **Supplementary Figure S6**).

### Adverse Events

Adverse events of capecitabine for TNBCs treatment were reported in four RCTs, which regarded TNBCs as the whole cohort (12, 17, 19, 24). We extracted data from the Grades 3–5 adverse events, which included neutropenia, leukopenia, diarrhea, fatigue, hand–foot syndrome, neuropathy, nail toxicity, stomatitis, and nausea. However, due to insufficient information or high heterogeneity, we only included six types of adverse events for further analysis (diarrhea, fatigue, hand–foot syndrome, neutropenia, leukopenia, and nausea). From the available data of TNBCs, the capecitabine group demonstrated significantly higher rates of diarrhea (OR = 3.10, 95% CI 2.32–4.15), hand–foot syndrome (OR = 25.79, 95% CI 15.32–43.42), and leukopenia (OR =2.08, 95% CI 1.13–3.84). Detailed outcomes are presented in **Table 1**.

**Table 1:** The result of adverse event.

	RCTs	Patients		Evidence synthesis(OR 95%CI)	I^2^ (%)	p-value	Egger’s test	GRADE
		Capecitabine	control					
Diarrhea	7	3479	3429	3.07 [2.30, 4.11]	65	<0.0001^*^	0.0752^*^	**High**
Fatigue	6	3281	3245	1.08 [0.88, 1.32]	59	0.45	0.0022^*^	Very low
hand-foot syndrome	6	2219	2153	23.72 [14.46, 38.92]	44	<0.0001^*^	0.0161^*^	**High**
Neutropenia	5	2845	2820	1.07 [0.92, 1.24]	90	0.40	0.0052^*^	Very low
Leukopenia	2	554	560	2.37 [1.23, 4.55]	25	0.01^*^	0.2533^*^	**High**
Nausea	2	733	713	2.07 [0.67, 6.39]	36	0.21	0.2255^*^	**High**

^*^p-value ≤ 0.05.

## Discussion

The efficacy of combining capecitabine with neoadjuvant or adjuvant chemotherapy in treating TNBCs has been discussed in many studies with different conclusions (10, 12, 13, 17–24). The CREATE-X trial compared two groups of patients treated with chemotherapy with or without capecitabine and concluded that the use of capecitabine was effective for TNBCs in terms of DFS (DFS: HR=0.58, 95% CI 0.39–0.86) (18). However, the GEICAM 2003-10 trial reached the opposite conclusion. The TX-CEX group demonstrated a worse result in DFS than the T+CEF group (DFS: HR=1.19, 95% CI 0.70–2.04) (21). Recently, two updated RCTs, EA1131 and SYSUCC-001 trial, published their results on the mixture of capecitabine and chemotherapy. The SYSUCC-001 trial included 434 cases and reported an evident increase in terms of DFS and OS (DFS: HR=0.64, 95% CI 0.42–0.95; OS: HR=0.75, 95% CI 0.47–1.19) (17). The use of capecitabine in the EA1131 trial mildly increased the DFS without statistical significance (DFS: HR=0.94, 95% CI 0.55–1.61) (12).

Li et al. conducted a meta-analysis in 2021 to corroborate the positive effect of capecitabine in TNBCs (25). However, some of the included patients were diagnosed with hormone receptor- and/or HER-2-positive breast cancer, leading to higher heterogeneity and reduced quality of conclusion (25). Moreover, a meta-analysis published in 2021 by Huo et al. discussed capecitabine and early TNBCs, but two newly published RCTs (EA1131 and SYSUCC-001) were not included (9). Our meta-analysis focused on adding capecitabine to the neo-/adjuvant chemotherapy and its benefit on the survival outcome for TNBCs. We excluded the RCTs that focused on whole breast cancer instead of TNBC and included 11 updated RCTs targeting capecitabine treatment. Therefore, with a larger sample and more available subgroups, our meta-analysis would be a better guideline for capecitabine in early TNBCs.

Our study evaluated the addition of capecitabine to early-stage TNBC patients receiving standard neoadjuvant or adjuvant chemotherapy. It showed significant DFS and OS improvement with tolerable adverse events. In the subgroup analysis, capecitabine played a different role in different types of chemotherapy (see **Table 2**). We found the following: (1) lower dosage group of capecitabine demonstrated better survival outcomes than the higher dosage group; (2) the group with more cycles of capecitabine showed more DFS increase than the less cycles group; (3) DFS only significantly improved upon using capecitabine as adjuvant chemotherapy (HR=0.74, 95% CI 0.65–0.84), but not upon using capecitabine as the neoadjuvant chemotherapy (HR=0.92, 95% CI 0.71–1.19); and (4) two studies evaluated the efficacy of capecitabine in TNBCs with different Ki-67 that, in cases, Ki-67 <30% resulted in better survival outcomes (Ki-67<30%: HR=0.53, 95%CI 0.29–0.98; Ki-67 > 30%: HR=0.70, 95% CI 0.51–0.98). Previously, Pasquier et al. explained that chemotherapy with low-dose capecitabine might reduce the recurrence rate for women with TNBC by two mechanisms of metastasis: angiogenesis and immune escape (26). Based on these results, we concluded that a lower dosage and higher frequency of capecitabine might be recommended for TNBCs, especially for mild cases. Besides, three RCTs reported the relationship between the nodal status of TNBCs and the addition of capecitabine in the chemotherapy (17, 19, 24). However, the available data did not demonstrate a significant change in DFS between lymph-node-positive patients and the negative ones.

**Table 2:** Outcomes of subgroup analysis.

	RCTs	Evidence synthesis (HR 95% CI)	I^2^ (%)	p-value	Egger’s test	Grade
***Dosage***						
>1,000 mg	8	0.79 [0.69, 0.91]	12	0.0007*	0.0684^*^	**High**
<1,000 mg	3	0.69 [0.54, 0.88]	0	0.002*	0.6909^*^	**High**
Subgroup difference			0	0.32		
***The number of cycles***						
>6 cycles	7	0.72 [0.62, 0.83]	0	<0.0001*	0.5226*	**High**
<6 cycles	4	0.87 [0.66, 1.15]	40	0.33	0.1849*	Moderate
Subgroup difference			32	0.23		
***Menopausal status***						
Premenopausal	2	0.72 [0.50, 1.05]	0	0.09	0.8122*	Moderate
Postmenopausal	2	0.79 [0.60, 1.04]	54	0.09	0.1073*	Low
Subgroup difference			0	0.72		
***Lymph node***						
Positive	3	0.68 [0.52, 0.89]	0	0.005*	0.4367*	**High**
Negative	3	0.68 [0.50, 0.92]	41	0.01*	0.3964*	**High**
Subgroup difference			0	1.00		
***Ki-67, %_c_***						
>30%	2	0.53 [0.29, 0.98]	0	0.04*	0.5206*	**High**
<30%	2	0.70 [0.51, 0.98]	0	0.04*	0.7476*	**High**
Subgroup analysis			0	0.42		
***Neo/adjuvant chemotherapy***						
Neoadjuvant	3	0.92 [0.71, 1.19]	0	0.51	0.6755*	Moderate
Adjuvant	9	0.74 [0.65, 0.84]	16	<0.0001*	0.0736*	**High**
Subgroup difference			52.7	0.15		

*p-value ≤ 0.05.

Capecitabine has been reported as effective adjuvant chemotherapy for gastric cancer and advanced breast (27, 28). After absorption, it would metabolize in the liver and cancerous tissues and finally convert into fluorouracil with the catalysis of thymidine phosphorylase (an enzyme rich in the breast) (29). A previous study confirmed that several doublets of cytotoxic agents, such as capecitabine and taxane, had synergistic activity *in vitro*, leading to better clinical benefits (30). It suggested that taxane could induce the intratumoral activity of thymidine phosphorylase and enhance the efficacy of capecitabine (30). Moreover, some researchers suggested that the efficacy of capecitabine might be associated with its long-term effects on dormant tumor cells, activating anti-cancer immunity or antiangiogenic activity (31–34).

However, the use of capecitabine comes with certain adverse events. It increased the incidence of diarrhea, hand–foot syndrome, and leukopenia. Hand–foot syndrome was the most significant adverse event. It is a reversible and non-life-threatening clinical condition, but it can significantly affect the patient’s quality of life. Early recognition, patient education, and supportive measures can reduce its negative impact, and COX-2 inhibitors were the most promising agents to treat the hand–foot syndrome (35). In a previous study, two women died from cardiac causes potentially related to the clinical trial (36). Cardiotoxicity is a common adverse event of the fluoropyrimidine class of chemotherapeutic agents, and patients receiving fluoropyrimidines need to be monitored constantly for cardiac adverse events. However, the cardiotoxicity of capecitabine may be milder than infused fluorouracil (see **Table 1**).

Apart from capecitabine, there are various treatment options for TNBCs. Some authors recommended that the combination of anthracycline and taxane, rather than anthracycline alone, was the standard chemotherapy for early-stage TNBC (7, 37, 38). Previous studies had also confirmed that Bevacizumab, a monoclonal antibody targeting vascular endothelial growth factor A, had efficacy in improving pathological complete response (pCR) rates when added into the neoadjuvant chemotherapy for TNBCs, with increased risk of neutropenia (39). Moreover, in I-SPY 2 trial, the addition of veliparib and carboplatin into the standard neoadjuvant chemotherapy significantly increased the pCR rates in TNBCs. However, this combination inevitably increased the rate of thrombocytopenia, neutropenia, and anemia (40). Considering the efficacy and the adverse events, capecitabine can be an effective regimen for treating TNBCs.

However, our meta-analysis had some limitations. First, most chemotherapy regimens in included RCTs were different combinations of anthracyclines, taxanes, and cyclophosphamide, but there were still some other regimens, leading to increased heterogeneity. Second, TNBCs were regarded as a subgroup in some included RCTs, and some baseline information of the patients was missing. Lastly, different types of adverse events were reported, and the effect of adverse events may be biased due to the lack of available data.

## Conclusion

The addition of capecitabine to early-stage TNBC patients receiving standard adjuvant chemotherapy showed significant DFS and OS improvement with tolerable adverse events. The lower dosage and higher frequency of capecitabine combined with adjuvant chemotherapy demonstrated a better survival outcome.

## Data Availability Statement

The original contributions presented in the study are included in the article/**Supplementary Material**. Further inquiries can be directed to the corresponding authors.

## Ethics Statement

Ethical review and approval was not required for the study on human participants in accordance with the local legislation and institutional requirements. The patients/participants provided their written informed consent to participate in this study.

## Author Contributions

RL and HH are in charge of the main idea and is the guarantor of integrity of the entire clinical study. XX is in charge of the study concepts, design, manuscript preparation, and editing. PH and YZ searched databases independently. QC and SR screened the titles and abstracts and articles meeting inclusion criteria. SR and YZ independently extracted data from eligible articles and conducted data analysis. XX and QC independently assessed the quality of included studies. PH and SR are in charge of the language polishing and the grammar revision. All authors contributed to the article and approved the submitted version.

## Conflict of Interest

The authors declare that the research was conducted in the absence of any commercial or financial relationships that could be construed as a potential conflict of interest.

## Publisher’s Note

All claims expressed in this article are solely those of the authors and do not necessarily represent those of their affiliated organizations, or those of the publisher, the editors and the reviewers. Any product that may be evaluated in this article, or claim that may be made by its manufacturer, is not guaranteed or endorsed by the publisher.

## Supplementary Material

Supplementary Figure 1 Shows the subgroup of dosage.

Supplementary Figure 2 Shows the subgroup of Neo/adjuvant chemotherapy.

Supplementary Figure 3 Shows the subgroup of cycles of capecitabine.

Supplementary Figure 4 Shows the result of ki-67.

Supplementary Figure 5 Shows the result of lymph node.

Supplementary Figure 6 Shows the result of menopausal status.

## Abbreviations

TNBC, triple-negative breast cancer; HRs, hazard ratios; ORs, odds ratios; CI, confidence intervals; DFS, disease-free survival; OS, overall survival; RCTs, randomized clinical trials; ER, estrogen receptor; PR, progesterone receptor; HER-2, human epidermal growth factor receptor 2; RFS, recurrence-free survival; pCR, pathological complete response.
