# Supplementary figures and images for: Efficacy and Safety of Capecitabine for Triple-Negative Breast Cancer: A Meta-Analysis
Source: Front Oncol. 2022 Jul 7;12:899423. doi: 10.3389/fonc.2022.899423 (PMC9300946; doi:10.3389/fonc.2022.899423)

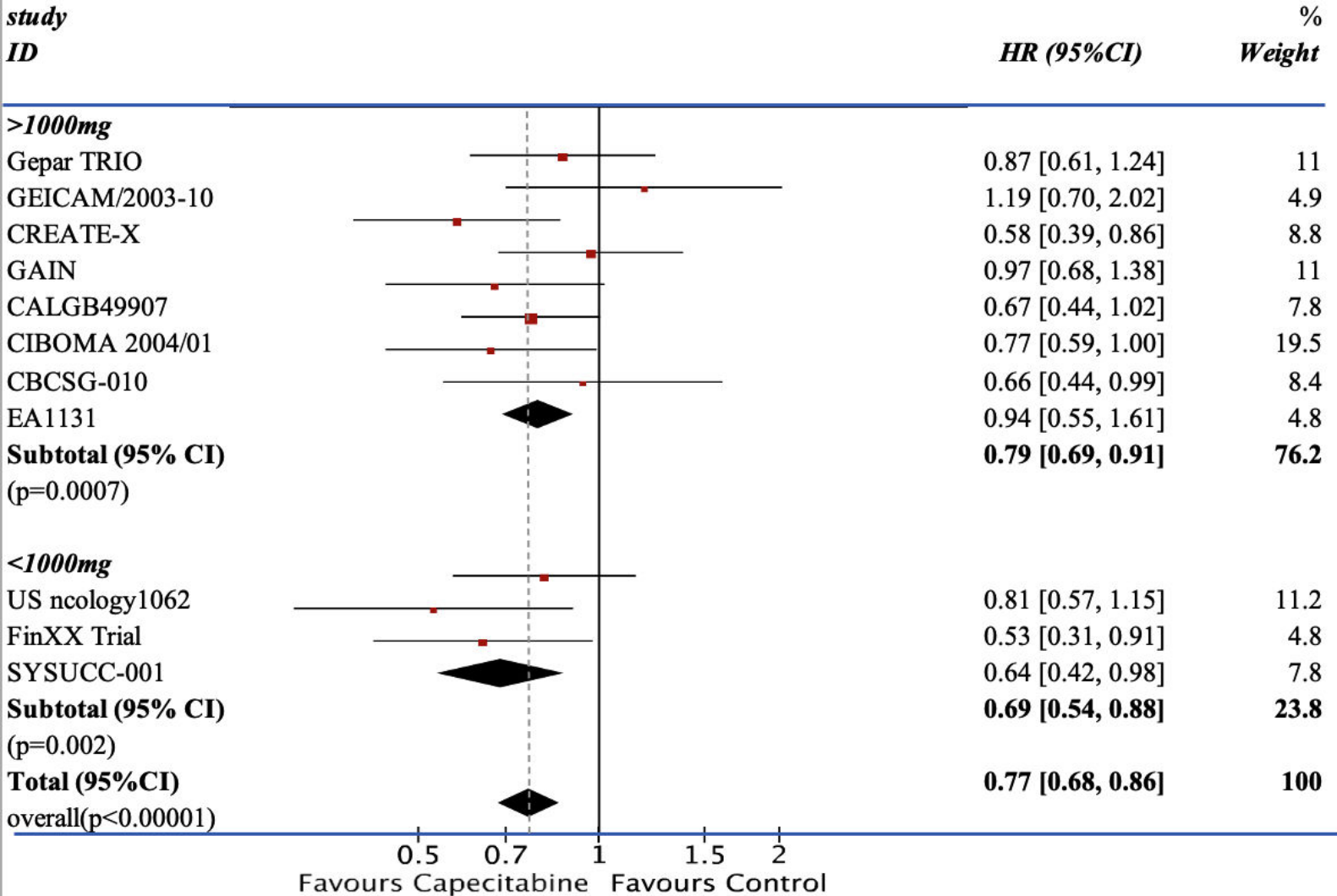

Supplement: Supplementary Figure 1 — Shows the subgroup of dosage. [file DataSheet_1.pdf]

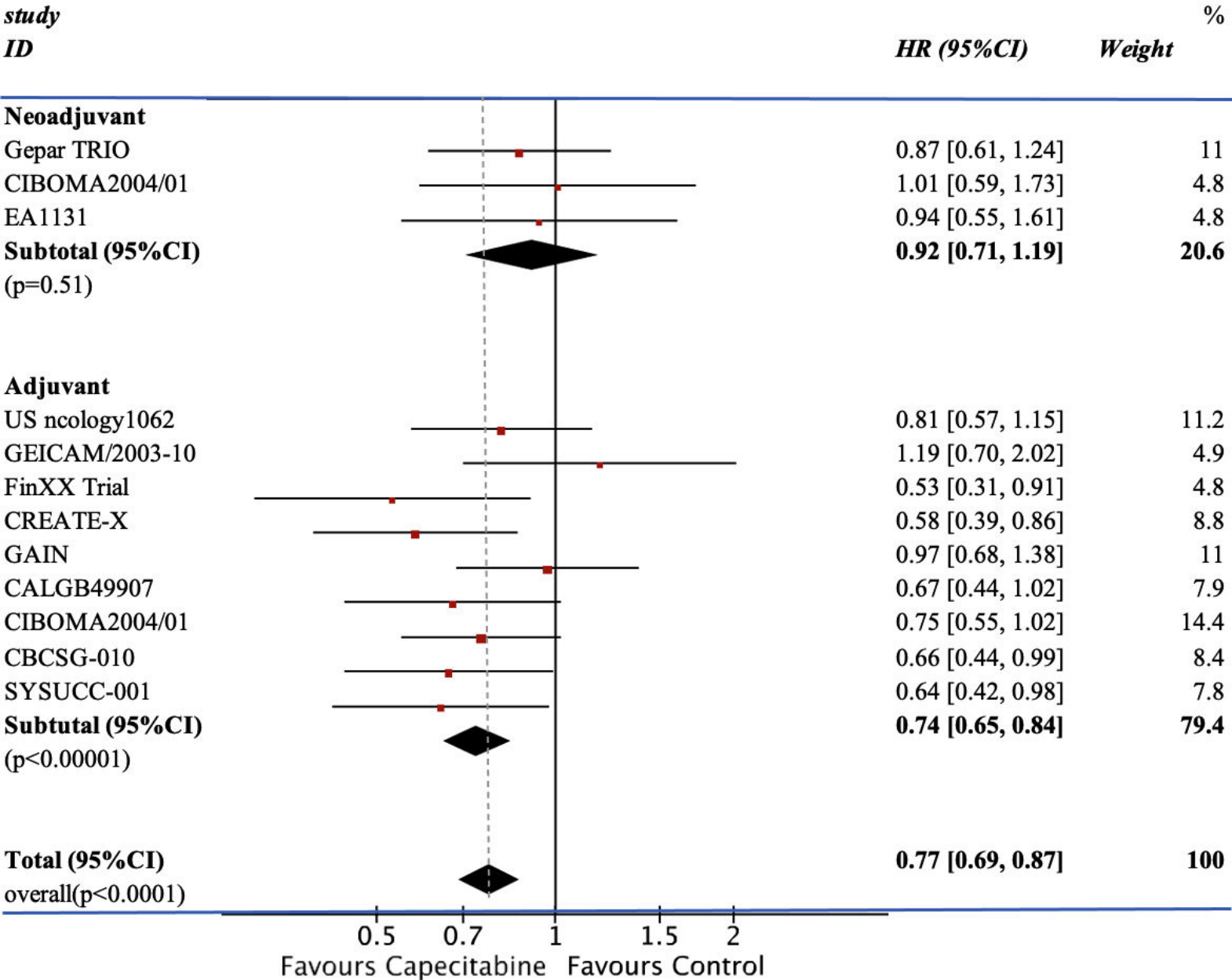

Supplement: Supplementary Figure 2 — Shows the subgroup of Neo/adjuvant chemotherapy. [file DataSheet_2.pdf]

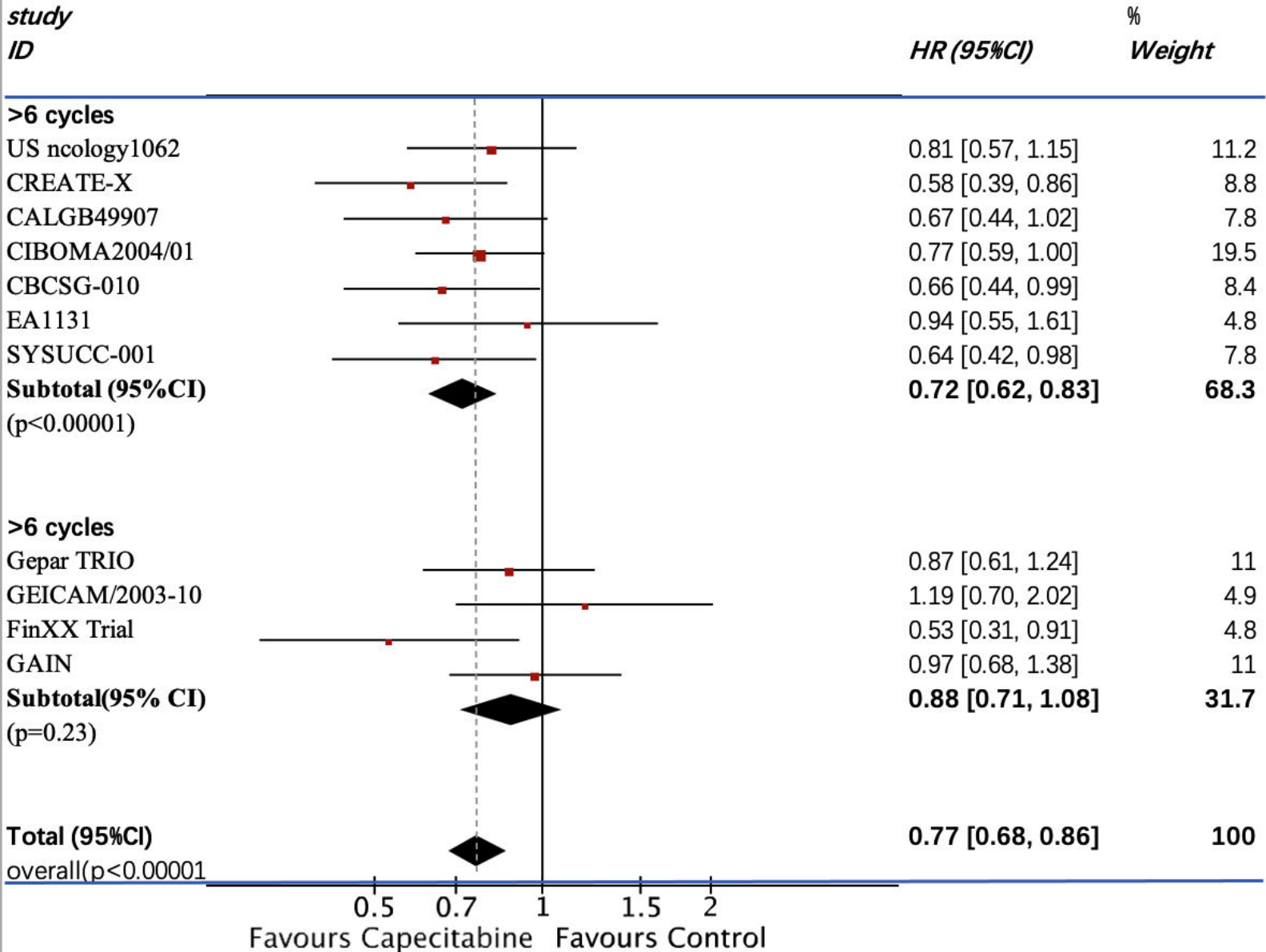

Supplement: Supplementary Figure 3 — Shows the subgroup of cycles of capecitabine. [file DataSheet_3.pdf]

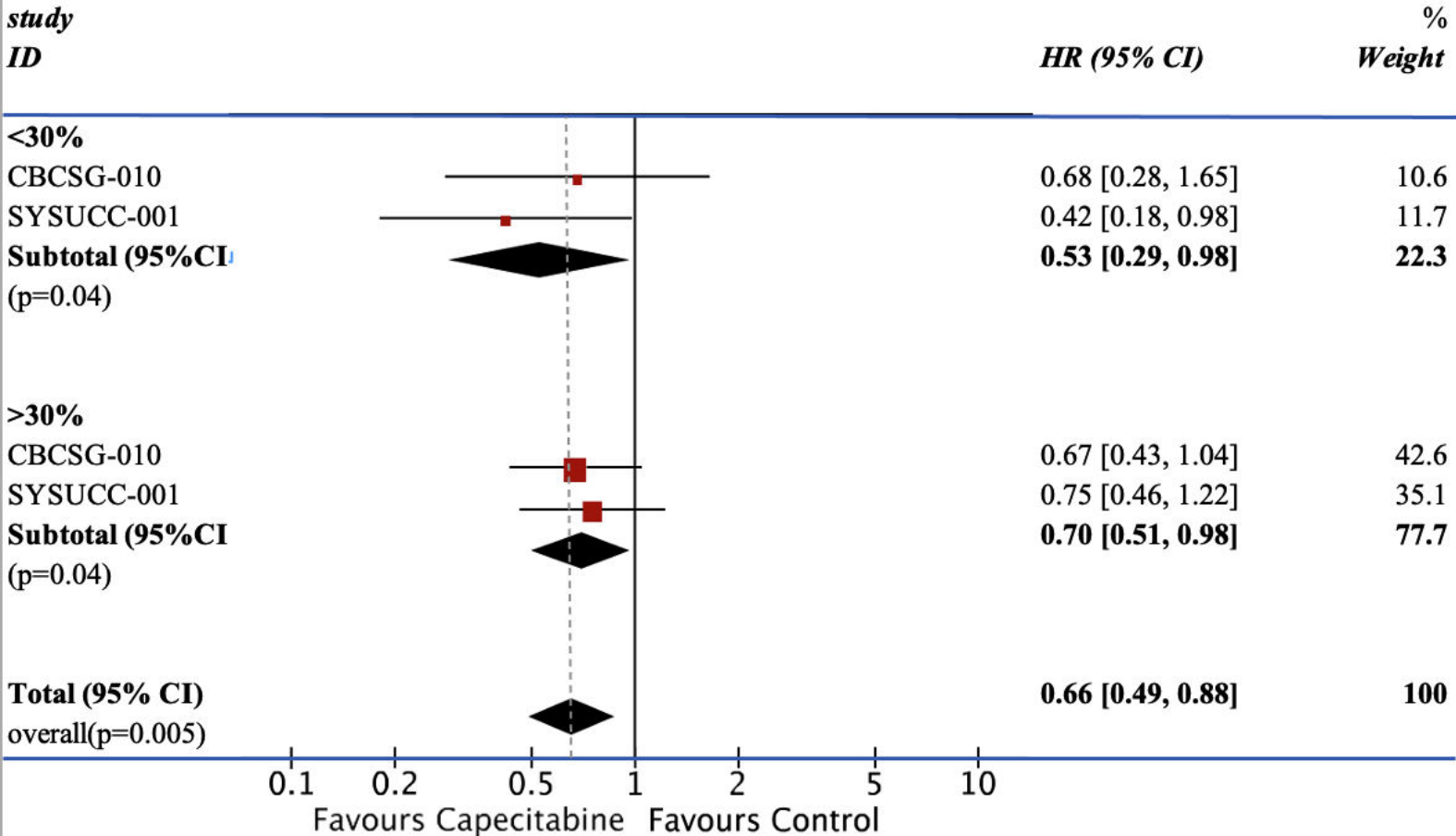

Supplement: Supplementary Figure 4 — Shows the result of ki-67. [file DataSheet_4.pdf]

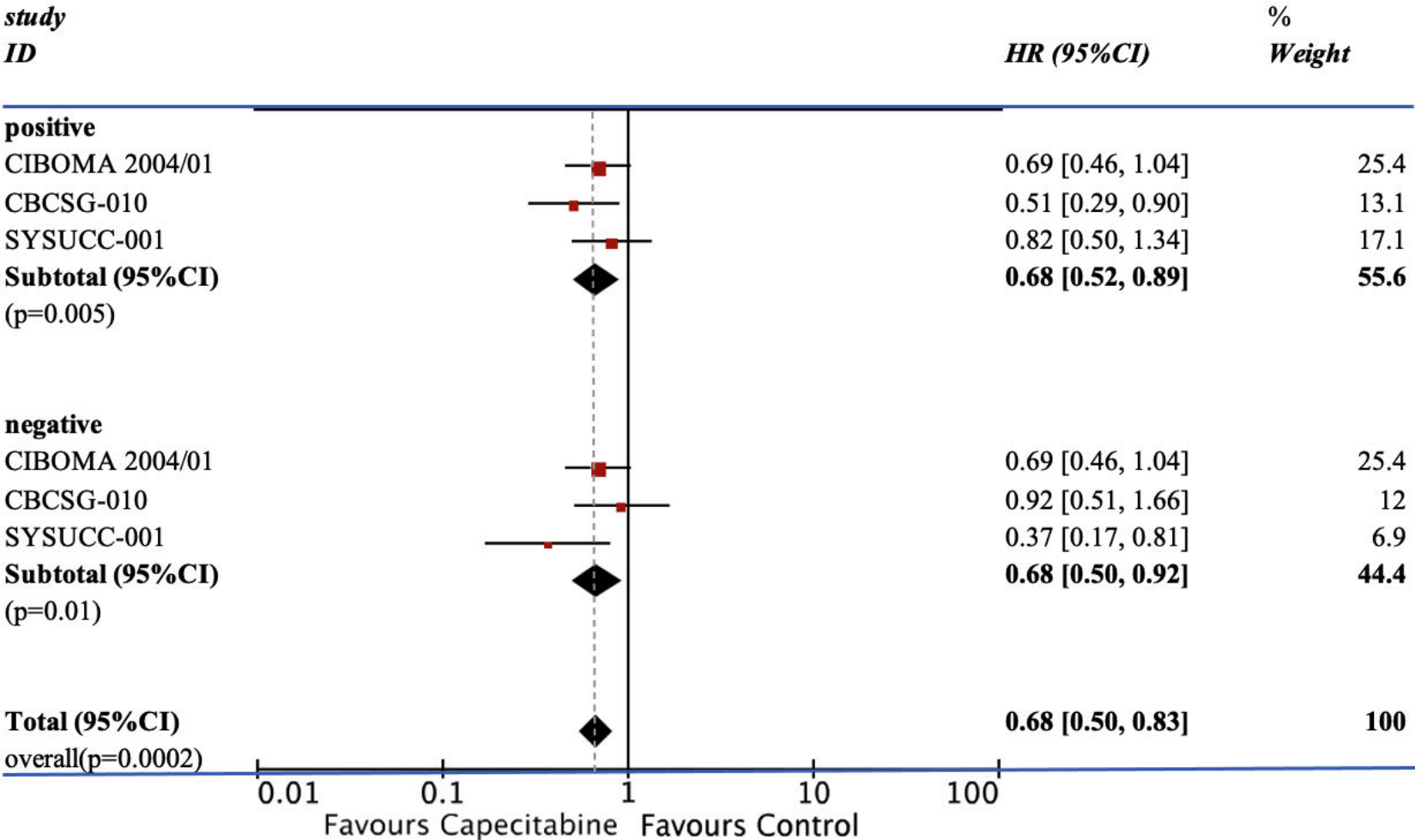

Supplement: Supplementary Figure 5 — Shows the result of lymph node. [file DataSheet_5.pdf]

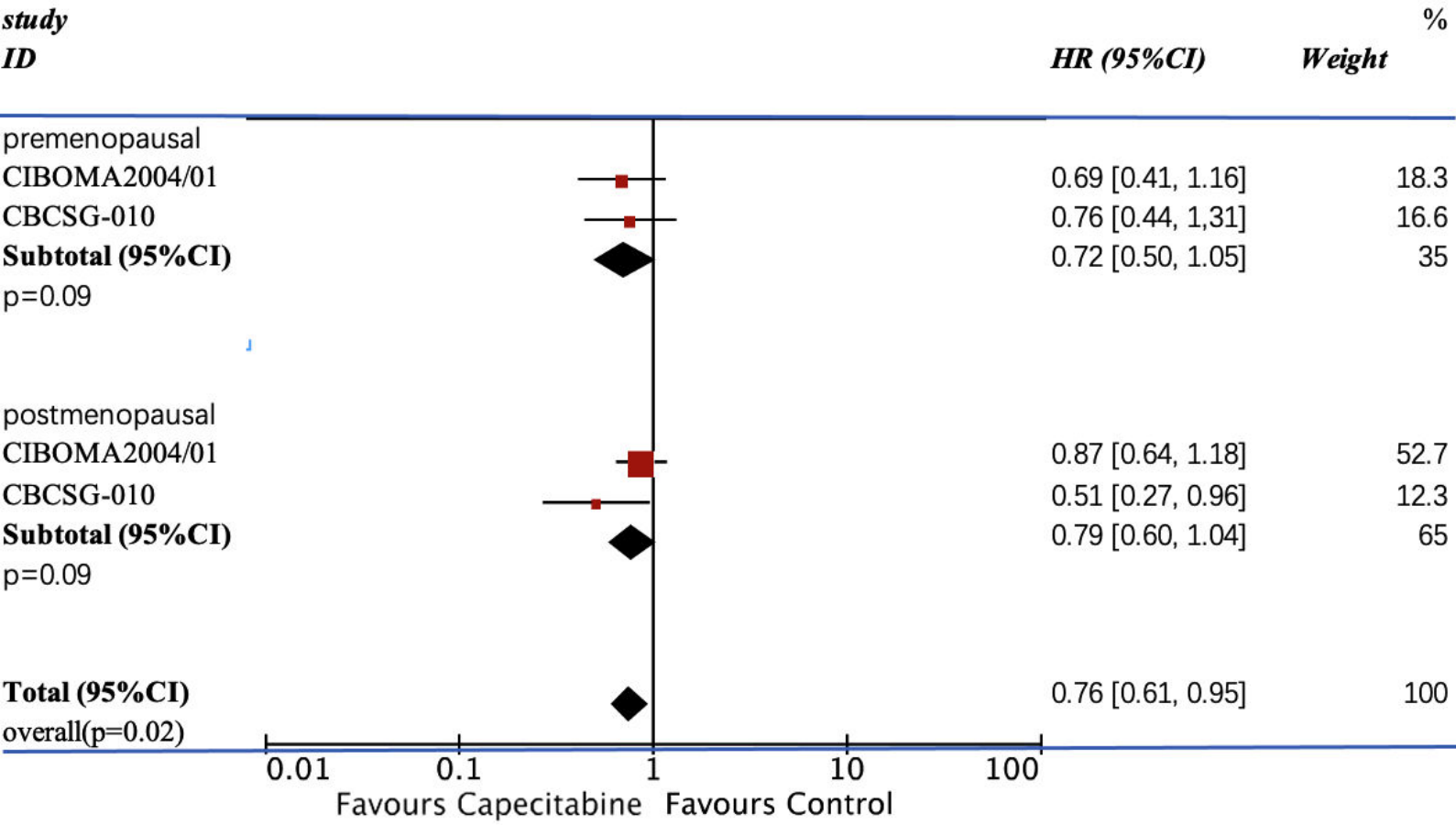

Supplement: Supplementary Figure 6 — Shows the result of menopausal status. [file DataSheet_6.pdf]
