# Supplementary material for: Efficacy and Safety of Capecitabine for Triple-Negative Breast Cancer: A Meta-Analysis
Source: Front Oncol. 2022 Jul 7;12:899423. doi: 10.3389/fonc.2022.899423 (PMC9300946; doi:10.3389/fonc.2022.899423)
Supplement: Supplementary file 7 [file Table_1.doc]

Table S1. Characteristics of studies included in systematic review

| Author Year | Study ID | Number | Exposure^#^ | Comparator | Age | Follow-up (months) | Funding |
| --- | --- | --- | --- | --- | --- | --- | --- |
| Mayer 2021 | EA1131 | 166 | ﻿Capecitabine group  (X) (n=95) | ﻿Control group (Pla) (n=71) | Capecitabine group:  51 (27-73) Control group:  51 (25-73) | 20 | ﻿US National Cancer Institute |
| Xi 2021 | SYSUCC-001 | 434 | ﻿Capecitabine group  (X)  (n=221) | Control group  (None)  (n=213) | ﻿Capecitabine group:  ﻿45 (39-53) ﻿ Control group：  ﻿48 (40-57) | 61 | Sun Yat-sen University Clinical Research 5010 Program |
| Joensuu 2017 | FinXX Trial | 202 | Capecitabine group  (TX-CEX) (n=93) | Control group (T+CEF)  (n=109) | Capecitabine group:  53 (45-59) Control group:  54 (44-60) | 123.6 | Finnish Breast Cancer  Group |
| Masuda 2017 | CREATE-X | 887 | ﻿Capecitabine group  (X)  (n=443) | ﻿ Control group (None)  (n=444) | Capecitabine group:  48 (25-74)  Control group：  48 (25-74) | 43.2 | the Advanced Clinical Research Organization and the Japan Breast Cancer Research Group |
| Lluch 2020 | CIBOMA 2004/01 | 876 | Capecitabine group  (ET-X) (n=448) | Control group  (EC-T) (n=428) | capecitabine group:  50 (20-79) control group:  49 (23-82) | 87.6 | F Hoffmann-LaRoche. |
| Muss 2019 | CALGB49907 | 154 | Capecitabine group  (X) (n=76) | Control group  (CMF/AC)  (n=78) | >65 | 136.8 | National Cancer Institute of the National Institutes  of Health |
| Li 2020 | CBCSG-010 | 585 | Capecitabine group  (TX-CEX)  (n=297) | Control group  (T-CEF)  (n=288) | Capecitabine group：﻿  49.1 ± 10.4﻿﻿ Control group：  ﻿﻿48.3 ± 8.7 | 67 | ﻿Jiangsu Hengrui Medicine Co, Ltd (Inst) |
| Martín 2015 | GEICAM/2003-10 | 166 | Capecitabine group  (ET-X)  (n=95) | Control group  (EC-T)  (n=71) | Capecitabine group:  51 (27-73) Control group:  51 (25-73) | 79.2 | ﻿Novartis |
| Moebus 2017 | GAIN | 421 | Capecitabine group  (EC-PX)  (n=213) | Control group  (EPC)  (n=208) | Not mentioned | 74 | ﻿Amgen Germany and  Roche Germany |
| O’Shaughnessy 2015 | US ncology 1062 | 780 | Capecitabine group  (AC-TX)  (n=396) | Control group  (AC-T)  (n=384) | Capecitabine group:  50 (26-72) Control group:  51 (26-70) | 60 | ﻿Hoffmann-La Roche Inc |
| Minckwitz 2013 | Gepar TRIO | Not mentioned | Capecitabine group  (TAC-NX)  (n=362) | Control group  (TAC-TAC)  (n=not mentioned) | Not mentioned | 62 | ﻿Amgen Germany and  Roche Germany |

| ^#^X: Capecitabine; C: cyclophosphamide; M: methotrexate; F: 5-fluorouracil; A: doxorubicin; E: eqirubicin; T: docetaxel; Pla:﻿ Platinum |  |
| --- | --- |
|  |
