# Supplementary material for: Efficacy and Safety of Capecitabine for Triple-Negative Breast Cancer: A Meta-Analysis
Source: Front Oncol. 2022 Jul 7;12:899423. doi: 10.3389/fonc.2022.899423 (PMC9300946; doi:10.3389/fonc.2022.899423)
Supplement: Supplementary file 8 [file Table_2.doc]

Table S2. Search Strategy of Medline

| Concept | Strategy | Result^#^ |
| --- | --- | --- |
| 1 | exp breast cancer / or breast neoplasms / | 322594 |
| 2 | (breast cancer * or breast neoplasms *).ti.ab | 10400 |
| 3 | 1 or 2 | 324234 |
| 4 | capecitabine *.ti,ab | 7105 |
| 5 | Xeloda *.ti.ab | 311 |
| 6 | 4 or 5 | 7157 |
| 7 | adjuvant chemotherapy*.ti.ab | 28786 |
| 8 | 3 AND 6 AND 7 | 76 |
| 9 | (clinical trial or randomized controlled trial). pt. | 932600 |
| 10 | 8 AND 9 | 31 |

^#^updated to Mar 18^th^ 2022

((((breast cancer [MeSH Terms]) OR (breast neoplasms [MeSH Terms])) OR ((breast cancer Title/Abstract]) OR (breast neoplasms [Title/Abstract]))) AND ((capecitabine [Title/Abstract]) OR (Xeloda [Title/Abstract])) AND (adjuvant chemotherapy [Title/Abstract])) AND ((clinical trial[Publication Type]) OR (randomized controlled trial[Publication Type]))
