# Supplementary material for: Efficacy and Safety of Capecitabine for Triple-Negative Breast Cancer: A Meta-Analysis
Source: Front Oncol. 2022 Jul 7;12:899423. doi: 10.3389/fonc.2022.899423 (PMC9300946; doi:10.3389/fonc.2022.899423)
Supplement: Supplementary file 9 [file Table_3.doc]

**Table S3. Assessment tools in meta-analysis**

**Cochrane Risk Of Bias Assessment Tool (CROBAT)**

| Random sequence generation | Allocation concealment | Blinding of participants and personnel | Blinding of outcome assessment | Incomplete outcome data | Selective reporting | Other bias |
| --- | --- | --- | --- | --- | --- | --- |
|  |  |  |  |  |  |  |
|  |  |  |  |  |  |  |

Each question had 3 answers: “Low risk”, “Moderate” and “High risk”.

“Low risk” when detailed methods were founded in manuscript.

“Moderate” when declared blinded without detailed method.

“High risk” when found no relevant declaration.

**Grading of Recommendations, Assessment, Development and Evaluation (GRADE)**

| Risk of  Bias | Inconsistency | Indirectness | Imprecision | Publication bias | Plausible Confounding | Magnitude of effect | Dose-response gradient |
| --- | --- | --- | --- | --- | --- | --- | --- |
|  |  |  |  |  |  |  |  |
|  |  |  |  |  |  |  |  |

Each following question had 3 answers: “No serious risk”, “Serious risk” and “Very serious risk”.

Risk of Bias: “Serious risk” when sensitive analysis resulted in significant difference.

Inconsistency: “No serious risk” when I^2^≤50%; “Serious risk”when 50<I^2^≤75%; “Very serious risk” when I^2^>75%

Indirectness: Comprehensive consideration in combination with information.

Imprecision: “Serious risk” when P>0.05

Publication bias: “No serious risk” when Egger’s test P>0.05; “Serious risk”when 0.01<P≤0.05; “Very serious risk” when P≤0.01.

Plausible Confounding had 2 answers: “No” and “Yes”. It would be assessed by comprehensive consideration in combination with information.

Magnitude of effect had 3 answers: “No”, “Yes” and “Extremely” related to odds ratio (OR). “No” when 0.5<OR<2; “Yes” when 0.2<OR≤0.5 or 2≤OR<5; “Extremely” when OR≤0.2 or OR≥5.

Dose-response gradient had 2 answers: “No” and “Yes”. “Yes” when P value of dose-response related outcome ≤0.05.

There were 4 levels of quality: “High”, “Moderate”, “Low” and “Very low”. Evidence of RCTs were initially assessed as “High”. “Serious risk” would reduce 1 level of quality and “Very serious risk” would reduce 2 levels of quality. While “Yes” could promote 1 level of quality and “Extremely” could promote 2 level of quality.
