# Supplementary material for: Efficacy and Safety of Capecitabine for Triple-Negative Breast Cancer: A Meta-Analysis
Source: Front Oncol. 2022 Jul 7;12:899423. doi: 10.3389/fonc.2022.899423 (PMC9300946; doi:10.3389/fonc.2022.899423)
Supplement: Supplementary file 10 [file Table_4.doc]

Table S4. Outcome of CROBAT assessment

| Author Year | Trail Name | Random sequence generation | Allocation concealment | Blinding of participants and personnel | Blinding of outcome assessment | Incomplete outcome data | Selective reporting | Other bias |
| --- | --- | --- | --- | --- | --- | --- | --- | --- |
| Mayer 2021 | EA1131 | Simple Random Sampling. | ﻿Capecitabine 1,000 mg/m2 twice daily, days 1-14, every 3 weeks, for a total of six cycles | Blinded | Blinded | No | No | No |
| Xi 2021 | SYSUCC-001 | Simple Random Sampling. | ﻿oral capecitabine at 650 mg/m2, twice daily continuously for 1 year | Blinded | Blinded | No | No | No |
| Joensuu 2017 | FinXX Trial | Simple Random Sampling. | 3 cycles of docetaxel plus capecitabine followed by 3 cycles of cyclophosphamide, epirubicin, and capecitabine | Blinded | Blinded | No | No | No |
| Masuda 2017 | CREATE-X | Simple Random Sampling. | oral capecitabine (at a dose of 1250 mg per square meter of body-surface area,twice per day, on days 1 to 14) every 3 weeks for six or eight cycles. | Blinded | Blinded | No | No | No |
| Lluch 2020 | ﻿GEICAM/2003-11_CIBOMA/2004-01 | Simple Random Sampling. | eight cycles of capecitabine 2,000 mg/m2(1,000 mg/m2administered orally two times per day) on days 1 to 14 every3 weeks | Blinded | Blinded | No | No | No |
| Muss 2019 | CALGB49907 | Simple Random Sampling. | a dosage of 2,000 mg/m2 per day for 14 consecutive days every 3 weeks for six cycles | Blinded | Blinded | No | No | No |
| Li 2020 | CBCSG-010 | Simple Random Sampling. | ﻿capecitabine plus docetaxel (XT: capecitabine 1,000 mg/m2 twice daily by ﻿mouth, days 1-14; docetaxel 75 mg/m2 as a 1-hour intravenous infusion on day 1 of every 3-week cycle) for 3 cycles, followed by capecitabine, epirubicin, and cyclophosphamide (XEC:capecitabine 1,000 mg/m2 twice daily,days 1-14; epirubicin 75 mg/m2 and cyclophosphamide 500 mg/m2 on day 1; every 3-week cycle) for 3 cycles. | Blinded | Blinded | No | No | No |
| Martín 2015 | GEICAM/2003-10 | Simple Random Sampling. | ﻿four cycles of epirubicin (90 mg/ m2) plus docetaxel (75 mg/m2) administered on day 1 every 3 weeks followed by four cycles of capecitabine (1,250 mg/m2twice a day on days 1 to 14). | Blinded | Blinded | No | No | No |
| Moebus 2017 | GAIN | Simple Random Sampling. | ﻿ddEC (E 112.5mg/m² + C 600mg/m², i.v. q2w for 4 cycles) followed by paclitaxel weekly (Pw 67.5mg/m² i.v. q8d for 10 weeks) plus capecitabine (X 2000mg/m² p.o. day 1-14, q22 for 4 cycles) (ddEC-PwX-regimen) | Blinded | Blinded | No | No | No |
| O’Shaughnessy 2015 | ﻿US ncology1062 | Simple Random Sampling. | ﻿8 cycles of AC--XT (four 3-weekly cycles of AC, then four 3-weekly cycles of X 825 mg/m2 orally twice daily on days 1 to 14 plus T 75 mg/m2 IV on day 1) | Blinded | Blinded | No | No | No |
| Minckwitz 2013 | ﻿Gepar TRIO | Simple Random Sampling. | ﻿two cycles of docetaxel 75 mg/m2, doxorubicin 50 mg/m2, and cyclophosphamide 500 mg/m2 (TAC) four cycles of vinorelbine 25 mg/m2 on days 1 and 8 plus capecitabine 1,000 mg/m2 orally twice a day on days 1 through 14, every 3 weeks (NX) | Blinded | Blinded | No | No | No |
